# Diversity of *Campylobacter* species in a rhesus macaque breeding colony

**DOI:** 10.1128/msphere.00991-24

**Published:** 2025-03-25

**Authors:** Irving Nachamkin

**Affiliations:** 1 Department of Pathology and Laboratory Medicine, Perelman School of Medicine, University of Pennsylvania 192716 , Philadelphia, Pennsylvania, USA; University of Michigan-Ann Arbor, Ann Arbor, Michigan, USA

**Keywords:** *Campylobacter*, rhesus macaques, spiral bacterial

## LETTER

The recent article by Bacon and colleagues (1) focused on the prevalence of *Campylobacter jejuni* and *Campylobacter coli* colonization in a population of rhesus macaques. It is useful to point out that other *Campylobacter* species as well as related spiral bacterial species such as *Helicobacter* spp. and *Arcobacter* spp. may also affect rhesus macaque health. A few studies have reported other *Campylobacter* species in this population including *Campylobacter fetus* subsp. *venerealis* (2) and *Campylobacter hyointestinalis* (3). *Helicobacter pylori* is well known to be associated with gastritis in these animals (4). *Helicobacter suis* was reported in a colony of *Macaca mulatta* without clear clinical significance (4). *Arcobacter* spp. have been rarely described in these nonhuman primates. *A. butzleri*, a known human pathogen, was described as a cause of diarrheal illness in rhesus macaques (5, 6).

Additional approaches to understand the prevalence of these spiral bacteria would be useful for population studies. The authors used a selective culture media, Campy Blood Agar (Blaser formulation) with antibiotics (cephalosporins) incubated in microaerobic conditions at 41℃–43°C which preferentially selects for thermophilic species, *Campylobacter jejuni* and *Campylobacter coli*. Different media formulations are better suited for isolating other *Campylobacter* species, such as a charcoal-based medium and other formulations with better performance characteristics, incubated at 37°C along with a microaerobic environment with increased H_2_ concentration (7). It should be noted that commercial gas packs used to generate microaerobic conditions generally have little to no hydrogen produced (7) so other sources to increase H_2_ would be required. Additionally, some *Campylobacter* species are susceptible to antibiotics in certain media formulations. Another method, such as a filtration method, may be better suited for isolating antibiotic-susceptible *Campylobacter* species (7). *Arcobacter* species are aerotolerant and may be recovered on some *Campylobacter* selective media and media designed for isolating *Arcobacter* species (7). *Helicobacter pylori* and other *Helicobacter* species are quite fastidious, require increased H_2_, and prolonged incubation for successful isolation on culture media (8).

In addition to culture-based methods, molecular approaches to survey nonhuman primate populations would be revealing of the spectrum of species commonly colonizing these non-human primates. Rhoades et al. (3) used metagenomic analysis to study the microbiomes of infant macaques and detected a number of unculturable bacterial species as well as *Campylobacter hyointestinalis*, a species that requires increased hydrogen for isolation in culture. Using broad-range 16S rDNA PCR methods on fecal material may reveal common or uncommon *Campylobacter* species (9) and *Helicobacter* species (10).

Additional comprehensive surveys using alternative culture methods and molecular approaches would add to our knowledge about the importance of other *Campylobacter* species and other spiral bacteria in health and disease of these nonhuman primates.
